# Public health round-up

**DOI:** 10.2471/BLT.21.011121

**Published:** 2021-11-01

**Malaria breakthrough**

Vaccinating an infant with the world’s first malaria vaccine as part of a pilot programme being implemented in Ghana, Kenya and Malawi that has reached more than 800 000 children since 2019. On 6 October, the World Health Organization recommended the vaccine for use among children to prevent *P. falciparum* malaria.

**Figure:**
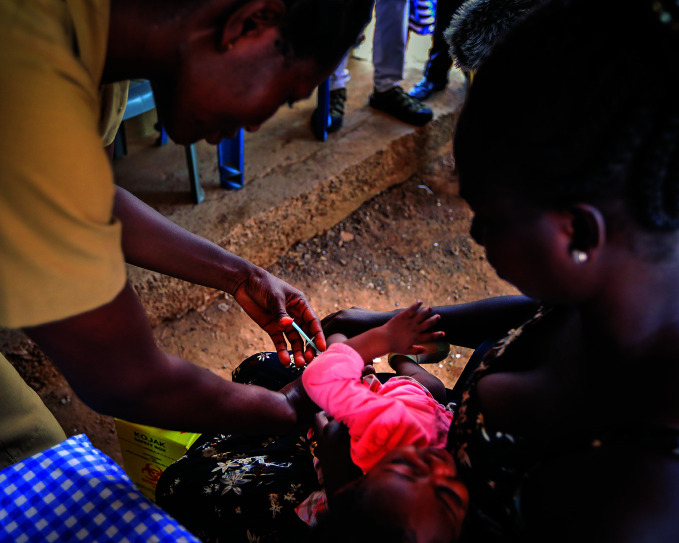
WHO/Fanjan Combrink

## First malaria vaccine

The World Health Organization (WHO) recommended widespread use of the first malaria vaccine among children in sub-Saharan Africa and in other regions with moderate to high *P. falciparum* malaria transmission.

Announced on 6 October, the recommendation is based on results from an ongoing pilot programme in Ghana, Kenya and Malawi that has reached more than 800 000 children since 2019.

The RTS,S/AS01 (RTS,S) vaccine is associated with a 30% reduction in severe malaria, even when introduced in areas where insecticide-treated nets are widely used and there is good access to diagnosis and treatment.

“Today’s recommendation offers a glimmer of hope for the continent which shoulders the heaviest burden of the disease and we expect many more African children to be protected from malaria and grow into healthy adults,” said Dr Matshidiso Moeti, WHO Regional Director for Africa.

https://bit.ly/2X9eGVi

## New COVID-19 treatment

WHO welcomed the addition of a new therapy to treat coronavirus disease 2019 (COVID-19). A combination of the monoclonal antibodies casirivimab and imdevimab, the therapy is associated with a substantial reduction in the relative risk of hospitalization of COVID-19 patients.

Because of the high cost and low availability of the combination therapy, UNITAID is negotiating with drug manufacturer Roche Pharmaceutical for lower prices and equitable distribution across all regions, especially in low- and middle-income countries. WHO is also in discussions with the company regarding possible donation and distribution of the drug through the United Nations Children’s Fund, following allocation criteria set by WHO.

In parallel, WHO launched a call to manufacturers who may wish to submit their products for pre-qualification, which would facilitate the ramping up of production.

https://bit.ly/3iVG68y

## Ebola in the Democratic Republic of the Congo

The Ministry of Health of the Democratic Republic of the Congo announced the detection of a new case of Ebola virus disease infection in the health zone of Butsili in North Kivu Province, where a previous outbreak was declared to have ended on 3 May 2021.

The Goma branch of the National Institute of Biomedical Research confirmed the presence of the virus in samples taken from a young child who died after suffering from Ebola-like symptoms on 6 October.

Butsili is close to Beni, a town which was one of the focuses of the 2018–2020 Ebola outbreak and is a commercial hub with links to the neighbouring countries of Uganda and Rwanda.

https://bit.ly/3oVfMPF

## Guinea Marburg outbreak ends

On 16 September 2021, the Ministry of Health of Guinea declared the end of the Marburg virus disease outbreak in Guéckédou prefecture, Nzérékoré Region. The declaration was made 42 days after the burial of the only confirmed patient reported in this outbreak and the first-ever Marburg virus disease case reported in Guinea.

During the outbreak, 173 contacts were identified, including 14 high-risk contacts based on exposure. Among them, 172 were followed for a period of 21 days, none developing symptoms. One high-risk contact was lost to follow-up. At the different points of entry in Guéckédou prefecture where passengers were screened, no alerts were generated.

Ongoing activities include the sampling of bats in the area to better understand their involvement in the ecology of Marburg viruses and development of a sero-surveillance protocol in the sub-prefecture of Koundou.

https://bit.ly/3lxsIJH

## Boosting COVID-19 vaccination rates

WHO launched a new strategy to boost COVID-19 vaccination rates. Released on 11 October, the strategy outlines a plan for achieving WHO’s targets to vaccinate 40% of the population of all countries worldwide by the end of 2021 and 70% by mid-2022.

To achieve the 40% target, shortfalls in the supply of vaccine to COVAX (the vaccine pillar of the Access to COVID-19 Tools Accelerator) need to be addressed immediately. The United Nations Secretary-General Antonio Guterres and WHO Director-General Tedros Adhanom Ghebreyesus called on countries and manufacturers to make good on their commitments without further delays.

In related news, on 16 September, a task force comprising the heads of the International Monetary Fund, the World Bank Group, WHO and the World Trade Organization met with the chief executive officers of leading vaccine manufacturing companies to discuss strategies to improve the access to COVID-19 vaccines, especially in low- and lower-middle-income countries. The task force expressed concerns that without urgent steps the world is unlikely to achieve the end-2021 target of vaccinating at least 40% of the population in all countries.

https://bit.ly/3iRDJUi

https://bit.ly/3mJjKsa

## Work-related disease and injury

Work-related diseases and injuries were responsible for the deaths of 1.9 million people in 2016. This is according to the first joint estimates from WHO and the International Labour Organization which were published on 16 September.

The greatest causes of deaths were estimated to be chronic obstructive pulmonary disease (450 000), stroke (400 000) and ischaemic heart disease (350 000). Occupational injuries were estimated to account for 360 000 deaths.

Key risk factors identified included long working hours, which were linked to approximately 750 000 deaths, and workplace exposure to air pollution (particulate matter, gases and fumes) which was assessed to be responsible for 450 000 deaths.

https://bit.ly/3jdfY9F

## Climate change and health

WHO released a special report on climate change and health in the lead-up to the United Nations Climate Change Conference (COP26) in Glasgow, Scotland. Launched on 11 October, the report is based on a growing body of research that establishes the multiple links between climate and health and sets out priority actions governments should take to tackle the climate crisis, restore biodiversity, and protect health. “It has never been clearer that the climate crisis is one of the most urgent health emergencies we all face,” said Dr Maria Neira, WHO Director of Environment, Climate Change and Health.

https://bit.ly/3mJthPS

## First global meningitis strategy

WHO and partners launched the first global strategy to defeat meningitis – a debilitating disease that kills hundreds of thousands of people each year. Launched on 28 September, the Global Roadmap to Defeat Meningitis by 2030 focuses on preventing infections and improving care and diagnosis for those affected, and sets targets for the elimination of epidemics of bacterial meningitis (the most deadly form of the disease), a 50% reduction in the total number of meningitis cases and a 70% reduction in meningitis deaths. The partners estimate that in total, the strategy could save more than 200 000 lives annually and significantly reduce disability caused by the disease.

https://bit.ly/3BEfXTa

## Updated essential medicines lists

WHO published new editions of its Model List of Essential Medicines and Model List of Essential Medicines for Children, which include new treatments for different cancers, insulin analogues and new oral medicines for diabetes, new medicines to assist people who want to stop smoking, and new antimicrobials to treat serious bacterial and fungal infections. The updated lists include 20 new medicines for adults and 17 for children and specify new uses for 28 already-listed medicines.

The listings aim to address global health priorities, identifying the medicines that provide the greatest benefits, and which should be available and affordable for all. High prices for both new, patented medicines and older medicines, like insulin, continue to keep some essential medicines out of reach for many patients.

https://bit.ly/2YF3hga

**Cover photo**

A member of a volunteer vaccination team marks a tally sheet during an immunization campaign in Awbarkhadle village, 20 kilometres east of Hargeisa, Somalia, on 20 August 2019.

**Figure:**
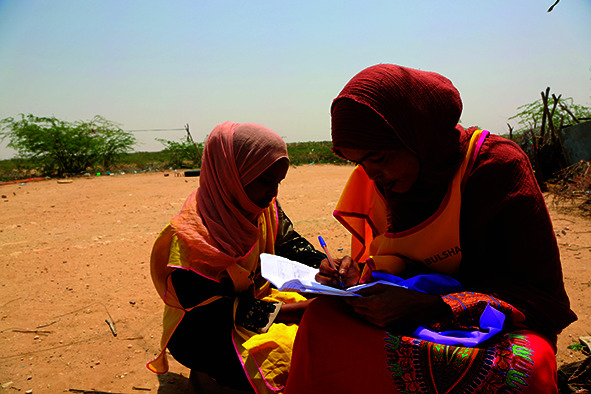
WHO/Ilyas Ahmed

## WHO Academy breaks ground

President of France, Emmanuel Macron, and WHO Director-General Tedros Adhanom Ghebreyesus broke ground for the WHO Academy’s campus in the French city of Lyon on 27 September.

The event marked a milestone in fulfilling a previous commitment by the two leaders to establish a WHO Academy in Lyon’s biomedical district to meet the needs of WHO Member States and a growing global health workforce for expanded access to lifelong learning, health guidance and competency-building.

https://bit.ly/30k9XBi

**Looking ahead**

31 October–12 November. United Nations Climate Change Conference of the Parties COP26. https://bit.ly/39hy1Gl

02–04 November. International Council of Nurses – 2021 Congress. https://bit.ly/3pbe5Oq

08–13 November. Ninth Session of the Conference of the Parties (COP9) to the WHO Framework Convention on Tobacco Control. https://bit.ly/3DLLtiO

